# Cottonseed extracts regulate gene expression in human colon cancer cells

**DOI:** 10.1038/s41598-022-05030-3

**Published:** 2022-01-20

**Authors:** Heping Cao, Kandan Sethumadhavan, Xiaoyu Wu, Xiaochun Zeng, Lin Zhang

**Affiliations:** 1 U.S. Department of Agriculture, Agricultural Research Service, Southern Regional Research Center, New Orleans, LA 70124 USA; 2 School of Bioscience and Bioengineering, Jiangxi Agricultural University, Nanchang, 330045 Jiangxi Province China; 3 Department of Life Science and Environmental Resources, Yichun University, Yichun, 336000 Jiangxi Province China; 4 Key Laboratory of Cultivation and Protection for Non-Wood Forest Trees, Ministry of Education, Central South University of Forestry and Technology, Changsha, 410004 Hunan Province China

**Keywords:** Biochemistry, Cancer, Plant sciences

## Abstract

Cotton plant provides economically important fiber and cottonseed, but cottonseed contributes 20% of the crop value. Cottonseed value could be increased by providing high value bioactive compounds and polyphenolic extracts aimed at improving nutrition and preventing diseases because plant polyphenol extracts have been used as medicinal remedy for various diseases. The objective of this study was to investigate the effects of cottonseed extracts on cell viability and gene expression in human colon cancer cells. COLO 225 cells were treated with ethanol extracts from glanded and glandless cottonseed followed by MTT and qPCR assays. Cottonseed extracts showed minor effects on cell viability. qPCR assay analyzed 55 mRNAs involved in several pathways including DGAT, GLUT, TTP, IL, gossypol-regulated and TTP-mediated pathways. Using BCL2 mRNA as the internal reference, qPCR analysis showed minor effects of ethanol extracts from glanded seed coat and kernel and glandless seed coat on mRNA levels in the cells. However, glandless seed kernel extract significantly reduced mRNA levels of many genes involved in glucose transport, lipid biosynthesis and inflammation. The inhibitory effects of glandless kernel extract on gene expression may provide a useful opportunity for improving nutrition and healthcare associated with colon cancer. This in turn may provide the potential of increasing cottonseed value by using ethanol extract as a nutrition/health intervention agent.

## Introduction

**Figure 1:**
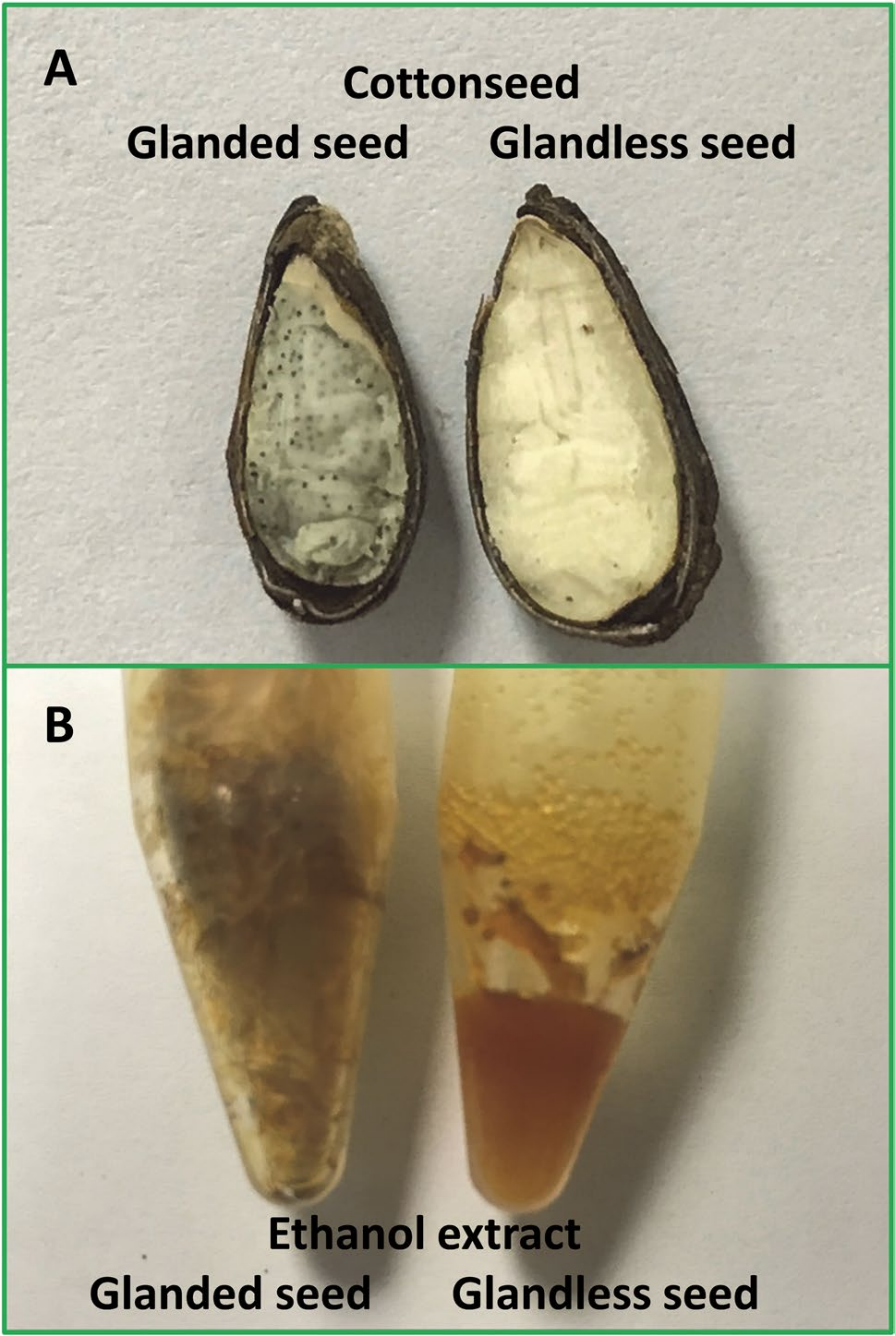
Cottonseed and ethanol extracts. (**A**) Glanded and glandless cottonseed section. Glanded seeds are smaller than glandless seeds and contain numerous dark-green-colored gossypol glands. (**B**) Ethanol extracts. Cottonseed coat or kernel was ground into fine powder and homogenized. The kernel fraction was defatted with chloroform and hexane. The coat fraction was treated with acetic acid followed by autoclave and centrifugation. The defatted materials were extracted with ethanol followed by evaporation to remove acetic acid and ethanol. Ethanol extracts were reconstitution in 100% DMSO (100 mg/mL) and analyzed by HPLC-MS^24^.

Cotton (*Gossypium hirsutum* L.) plant provides economically important fiber and cottonseed. Cottonseed contributes to approximately 20% of the crop value. It is either glanded or glandless depending on its seed with or without gossypol glands (Fig. 1A)^1–3^. Glanded cottonseed contains high concentrations of gossypol^4^, which limits its use primarily to feed ruminants due to its toxicity towards humans and most animals^5–9^. Glandless cottonseed has only trace levels of gossypol which may be useful as a food for humans or feed for non-ruminant animals^10–13^. Glanded and glandless cottonseed contains many other bioactive components including quercetin, gallic acid, 3,4-dihydroxybenzoic acid, flavonoids, cyclopropenoid fatty acids, and peptides. Most of these value-added products possess health promotion and disease prevention potentials^14,15^. Since plant bioactive products have been used for disease prevention and treatment since ancient history, cottonseed value could be increased by providing high value bioactive compounds and polyphenolic extracts aimed at improving nutrition and preventing diseases.

Colon cancer is a serious disease with 4.3% men and 4.0% women developing colorectal cancer during their lifetime according to American Cancer Society’s 2021 estimate (www.cancer.org). According to World Cancer Research Fund International, colorectal cancer is the third most commonly occurring cancer in men and the second most commonly occurring cancer in women. There were over 1.8 million new cases in 2018 (www.wcrf.org).

Plant polyphenols are major bioactive compounds present in most diet with beneficial effects on human health^16^. They regulate gene expression in numerous studies. Green tea polyphenols affect many gene expression in rats fed a high fructose diet^17,18^. Cinnamon polyphenols regulate the expression of genes involved in the insulin signaling pathway, inflammatory responses and lipid metabolism^19–23^. We recently isolated bioactive ethanol extracts from glanded and glandless cottonseed which were shown to be essentially free of gossypol by HPLC–MS analysis (Fig. 1B)^24^. These bioactive cottonseed extracts affect human cancer cell growth^24^. They also regulate mouse gene expression coding for diacylglycerol acyltransferase (DGAT), tristetraprolin/zinc finger protein 36 (TTP/ZFP36) family genes and human antigen R (HuR)^25–27^. However, cottonseed extracts on gene expression in cancer cells was unknown.

It is our aim to survey the effects of cottonseed ethanol extracts on regulating the expression of a wide range of genes involved in colon cancer cells. In this study, we analyzed the effects of cottonseed extracts on cell viability and expression of 55 genes which were shown to be regulated by cottonseed-derived gossypol in cancer cells^28–35^ and macrophages^26^ or by TTP/ZFP36 in tumor cells^36–44^ and macrophages^21,23^. The genes selected for analysis are involved in a variety of pathways including lipid biosynthesis (DGATs), glucose transport (GLUTs), anti-inflammation (TTP family), pro-inflammation (TNF, COX, CSF, HUA, ILs, VEGFs), cancer development (BCL2, BNIP3, CYP19A1, FAS, HUA, P53, PPARR and TNFSF10), and TTP-mediated mRNA stability (AHRR1, BCL2L1, CsnK2A1, CXCL1, E2F1, ELK1, HIF1a, HMOX1, ICAM1 and ZFAND5) (Table 1). Cottonseed extracts were used to treat human colon cancer cells (COLO 225) followed by MTT assay and quantitative PCR analysis. COLO 225 (ATCC CCL 222) was selected for the experiments because (1) it is derived from metastatic site with colorectal adenocarcinoma of human, (2) it is loosely attached to the surface of flasks for easy manipulation with trypsinization, and (3) it is widely used in cancer research as a cell model^45–52^. Our results showed that ethanol extracts from glandless cottonseed kernel significantly reduced the expression of many genes in the colon cancer cells.

**Table 1:** Human mRNA targets analyzed by qPCR; whose levels are regulated by cinnamon extract, gossypol or TTP as indicated in the “Reference” column.

ID	mRNA	Name	References
H1	Ahrr1	Aryl hydrocarbon receptor repressor	TTP^39^
H2	Bcl2	B-cell lymphoma 2	Gossypol^32^
H3	Bcl2l1	B-cell lymphoma 2 like 1	TTP^82^
H4	Bnip3	BCL2 protein-interacting protein 3	Gossypol^35^
H5	Cd36	Cluster of differentiation 36/fatty acid translocase	TTP^94^
H6	Claudin1	Maintain tissue integrity and water retention	TTP^44^
H7	Cox1	Cyclooxygenase 1	TTP^95^
H8	Cox2	Cyclooxygenase 2	TTP^43^
H9	Csnk2a1	Casein kinase 2 alpha 1	TTP^41^
H10	Ctsb	Cathepsin B	TTP^96^
H11	Cxcl1	Chemokine (C-X-C motif) ligand 1	TTP^83^
H12	Cyclind1	Cyclin D1	Gossypol^33^
H13	Cyp19a1	Cytochrome P450 family 19 subfamily A member 1	Gossypol^30^
H14	Dgat1	Diacylglycerol O-acyltransferase 1	Cinnamon^22,97^
H15	Dgat2a	Diacylglycerol O-acyltransferase 2a	Cinnamon^22,98^
H16	Dgat2b	Diacylglycerol O-acyltransferase 2b	Cinnamon^22,98^
H17	E2f1	E2F transcription factor 1	TTP^40^
H18	Elk1	ETS transcription factor	TTP^37^
H19	Fas	Fas cell surface death receptor	Gossypol^29^
H20	Gapdh	Glyceraldehyde-3-phosphate dehydrogenase	Reference^80^
H21	Glut1	Glucose transporter 1	Cinnamon^23^
H22	Glut2	Glucose transporter 2	Cinnamon^23^
H23	Glut3	Glucose transporter 3	Cinnamon^23^
H24	Glut4	Glucose transporter 4	Cinnamon^23^
H25	Hif1a	Hypoxia inducible factor 1 subunit alpha	TTP^84^
H26	Hmgr	3-Hydroxy-3-methylglutaryl-CoA reductase	^88^
H27	Hmox1	Heme oxygenase 1	TTP^85^
H28	Hua	Human antigen a	Gossypol^26^
H29	Icam1	Intercellular adhesion molecule 1/CD54	^86^
H30	Inos	Inducible nitric oxide synthase	^99^
H31	Insr	Insulin receptor	^21^
H32	Il2	Interleukin 2	TTP^63^
H33	IL6	Interleukin 6	TTP^64^
H34	IL8	Interleukin 8	TTP^65^
H35	Il10	Interleukin 10	TTP^66^
H36	Il12	Interleukin 12	TTP^67^
H37	Il16	Interleukin 16	TTP^42^
H38	Il17	Interleukin 17	TTP^68^
H39	Leptin	Body fat and obesity hormone	^81^
H40	Map1lc3a	Microtubule-associated proteins 1 light chain 3A	^89^
H41	Map1lc3b	Microtubule-associated proteins 1 light chain 3B	^89^
H42	Nfkb	Nuclear factor kappa B	^90^
H43	P53	Tumor suppressor	Gossypol^28^
H44	Pim1	Proto-oncogene serine/threonine-protein kinase	TTP^38^
H45	Pparr	Peroxisome proliferator-activated receptor gamma	Gossypol^31^
H46	Rab24	Ras-related oncogene 24	^100^
H47	Rpl32	Ribosomal protein L32 (60S ribosomal unit)	Reference^77^
H48	Tnf	Tumor necrosis factor	TTP^64^
H49	Tnfsf10	Tumor necrosis factor superfamily, member 10	Gossypol^34^
H50	Ulk2	Unc-51 like autophagy activating kinase 2	^101^
H51	Vegf	Vascular endothelial growth factor	TTP^36^
H52	Zfand5	Zinc finger AN1-type containing 5	TTP^87^
H53	Zfp36/Ttp	Zinc finger protein 36	TTP^21^
H54	Zfp36l1	Zinc finger protein 36 like 1	TTP^21^
H55	Zfp36l2	Zinc finger protein 36 like 2	TTP^21^

## Results

### Effect of cottonseed ethanol extracts on colon cancer cell viability

**Figure 2:**
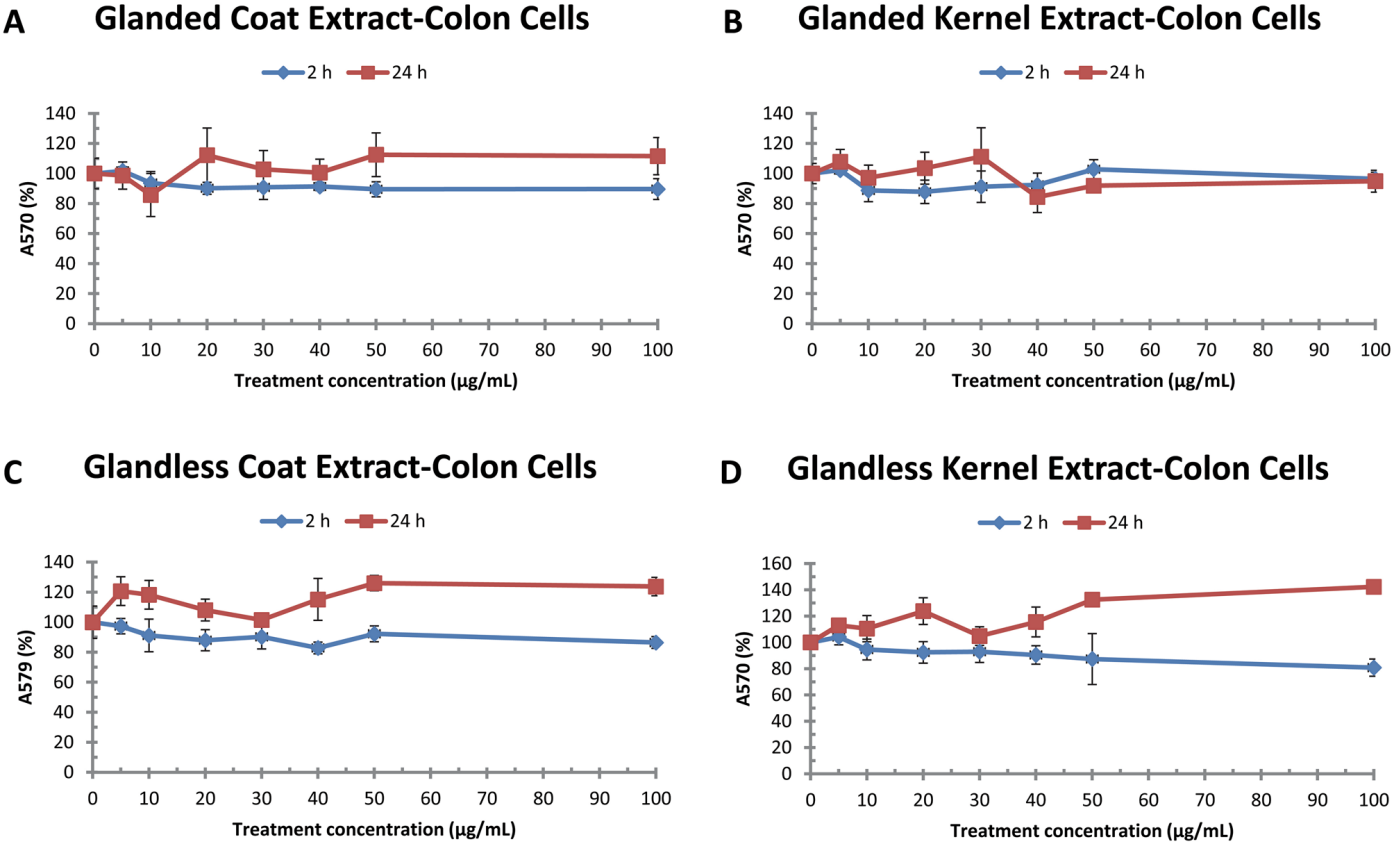
Effect of cottonseed extracts on human colon cancer cell viability. (**A**) Glanded cottonseed coat extract, (**B**) Glanded cottonseed kernel extract, (**C**) Glandless cottonseed coat extract, (**D**) Glandless cottonseed kernel extract. Colon cancer COLO 225 cells were treated with cottonseed extracts for 2 and 24 h. Cell viability was determined by MTT assay. The data represent the mean and standard deviation of three independent samples.

Before cottonseed extracts on gene expression were analyzed, we evaluated the effect of the ethanol extracts on colon cancer cell growth. Human colon cancer cells (COLO 225) were treated with 10–100 µg/mL of cottonseed extracts for 2 and 24 h. MTT assay was used to estimate the effect of cottonseed extracts on cell viability. MTT assay did not show significant changes in the viability of colon cancer cells under treatments with various concentrations for 2 or 24 h (Fig. 2). Similar analysis did not show major effect of these cottonseed extracts on the viability of human lung cancer cells (A549 CCL185) (data not shown). Colon cancer cells were selected for gene expression analysis as described below.

## Basal gene expression level in human colon cancer cells

One important factor for relative gene expression evaluation is to get a basic idea about the basal level expression of the genes selected for investigation. The relative mRNA levels of 55 genes (Table 1) were measured in the control cells using the specific qPCR primer pairs as described^53^. SYBR Green qPCR assay showed that BCL2 mRNA C_T_ (cycle of threshold) was one of the least varied mRNAs (Table 2). BCL2 mRNA C_T_ value was 30 ± 1 (mean ± standard deviation, n = 12) (Table 2). GAPDH and RPL32 mRNA levels were 33 and 51 fold of BCL2 mRNA, respectively. INOS mRNA was undetectable. AHRR1, COX1, CYCLIND1, GLUT4, HUA, ICAM1, IL10, IL12, RAB24, VEGF and ZFP36L2 mRNAs were detected with less than 10% of BCL2 mRNA in the colon cancer cells (Table 2).

**Table 2:** Basal level and reference mRNA selection.

ID	mRNA	DMSO control		Glanded coat		Glanded kernel		Glandless coat		Glandless kernel	
		Mean ± Std	Fold	Mean ± Std	Fold	Mean ± Std	Fold	Mean ± Std	Fold	Mean ± Std	Fold
H1	Ahrr1	34 ± 1	0.1	35 ± 1	0.0	34 ± 1	0.0	35 ± 1	0.0	32 ± 1	0.1
H2	Bcl2	30 ± 1	1.0	29 ± 1	1.0	29 ± 1	1.0	29 ± 1	1.0	28 ± 1	1.0
H3	Bcl2l1	28 ± 2	3.4	28 ± 2	2.4	28 ± 2	1.6	28 ± 2	1.7	27 ± 2	2.4
H4	Bnip3	28 ± 1	3.2	27 ± 1	2.7	27 ± 1	2.4	27 ± 1	2.9	26 ± 1	3.0
H5	Cd36	29 ± 1	1.9	29 ± 2	1.0	28 ± 1	1.4	28 ± 3	1.3	28 ± 1	1.6
H6	Claudin1	31 ± 7	0.5	31 ± 4	0.2	32 ± 5	0.1	31 ± 4	0.2	29 ± 5	0.7
H7	Cox1	40 ± 5	0.0	40 ± 3	0.0	39 ± 5	0.0	37 ± 3	0.0	38 ± 5	0.0
H8	Cox2	30 ± 2	0.7	29 ± 2	0.7	29 ± 2	0.5	29 ± 1	0.8	28 ± 1	1.0
H9	Csnk2a1	27 ± 2	9.7	27 ± 2	4.4	27 ± 2	3.5	27 ± 1	4.2	26 ± 2	6.3
H10	Ctsb	28 ± 3	3.2	29 ± 2	1.1	29 ± 2	0.7	29 ± 2	0.9	28 ± 3	1.7
H11	Cxcl1	31 ± 2	0.4	35 ± 5	0.0	36 ± 7	0.0	36 ± 6	0.0	35 ± 6	0.0
H12	Cyclind1	34 ± 7	0.1	33 ± 5	0.1	34 ± 6	0.0	32 ± 5	0.2	33 ± 7	0.0
H13	Cyp19a1	30 ± 1	1.0	29 ± 1	0.8	29 ± 1	0.7	29 ± 1	0.8	29 ± 1	0.7
H14	Dgat1	30 ± 2	1.1	30 ± 2	0.6	30 ± 2	0.5	30 ± 1	0.5	29 ± 2	0.5
H15	Dgat2a	33 ± 3	0.1	32 ± 2	0.1	32 ± 2	0.1	32 ± 2	0.1	31 ± 2	0.2
H16	Dgat2b	32 ± 2	0.3	31 ± 2	0.2	31 ± 2	0.1	31 ± 1	0.3	30 ± 1	0.3
H17	E2f1	30 ± 1	0.9	29 ± 1	0.8	29 ± 1	0.6	29 ± 1	0.9	29 ± 1	0.7
H18	Elk1	30 ± 3	0.6	32 ± 3	0.1	32 ± 3	0.1	32 ± 2	0.1	29 ± 1	0.6
H19	Fas	31 ± 6	0.4	31 ± 4	0.2	32 ± 5	0.1	30 ± 3	0.4	30 ± 5	0.3
H20	Gapdh	25 ± 5	32.5	25 ± 3	17.6	25 ± 3	10.3	25 ± 3	19.0	23 ± 3	39.3
H21	Glut1	27 ± 3	5.2	28 ± 4	1.3	28 ± 2	1.1	28 ± 2	2.3	27 ± 3	2.5
H22	Glut2	30 ± 2	0.8	29 ± 2	0.8	29 ± 2	1.0	29 ± 1	1.0	28 ± 1	1.2
H23	Glut3	28 ± 1	2.7	28 ± 1	2.4	27 ± 1	2.4	27 ± 1	3.0	27 ± 1	2.8
H24	Glut4	41 ± 6	0.0	39 ± 8	0.0	40 ± 9	0.0	40 ± 9	0.0	40 ± 8	0.0
H25	Hif1a	27 ± 2	5.7	28 ± 2	2.3	28 ± 2	1.9	28 ± 2	1.8	27 ± 2	3.7
H26	Hmgr	28 ± 2	3.9	28 ± 2	1.4	28 ± 2	1.6	28 ± 1	1.8	27 ± 2	3.0
H27	Hmox1	30 ± 1	0.7	29 ± 1	0.7	30 ± 1	0.4	30 ± 1	0.6	30 ± 3	0.3
H28	Hua	33 ± 5	0.1	32 ± 2	0.1	32 ± 2	0.1	32 ± 2	0.1	32 ± 4	0.1
H29	Icam1	35 ± 5	0.0	36 ± 6	0.0	34 ± 2	0.0	35 ± 4	0.0	34 ± 4	0.0
H30	Inos	ud		ud		ud		ud		ud	
H31	Insr	30 ± 4	1.0	32 ± 4	0.1	31 ± 3	0.1	31 ± 3	0.2	31 ± 5	0.2
H32	Il2	32 ± 1	0.2	31 ± 1	0.2	31 ± 1	0.2	31 ± 1	0.2	31 ± 1	0.2
H33	IL6	30 ± 1	0.9	29 ± 2	0.7	29 ± 1	0.9	29 ± 1	0.9	28 ± 2	1.3
H34	IL8	29 ± 1	1.5	29 ± 1	1.0	29 ± 1	0.9	29 ± 1	1.1	28 ± 1	1.0
H35	Il10	39 ± 5	0.0	35 ± 14	0.0	32 ± 14	0.1	30 ± 17	0.6	31 ± 15	0.2
H36	Il12	39 ± 3	0.0	39 ± 4	0.0	37 ± 3	0.0	37 ± 3	0.0	34 ± 2	0.0
H37	Il16	29 ± 1	2.1	29 ± 4	0.8	28 ± 1	1.1	29 ± 2	1.1	28 ± 1	1.6
H38	Il17	30 ± 1	0.7	30 ± 2	0.5	29 ± 1	0.6	30 ± 2	0.6	29 ± 1	0.7
H39	Leptin	31 ± 7	0.4	27 ± 10	3.9	31 ± 4	0.2	30 ± 5	0.4	27 ± 11	2.5
H40	Map1lc3a	30 ± 2	0.7	29 ± 1	0.7	30 ± 2	0.5	29 ± 1	0.8	29 ± 1	0.7
H41	Map1lc3b	27 ± 2	9.3	27 ± 2	3.2	27 ± 1	3.4	27 ± 1	4.1	26 ± 4	4.2
H42	Nfkb	31 ± 4	0.5	34 ± 4	0.0	39 ± 6	0.0	38 ± 5	0.0	33 ± 4	0.1
H43	P53	31 ± 3	0.4	32 ± 3	0.1	31 ± 2	0.1	31 ± 2	0.2	30 ± 2	0.3
H44	Pim1	30 ± 1	1.2	30 ± 2	0.6	29 ± 1	0.7	29 ± 1	0.8	29 ± 1	0.8
H45	Pparr	29 ± 1	1.4	29 ± 1	0.8	29 ± 1	0.7	29 ± 1	0.8	28 ± 1	0.9
H46	Rab24	42 ± 3	0.0	48 ± 1	0.0	42 ± 3	0.0	44 ± 4	0.0	42 ± 4	0.0
H47	Rpl32	24 ± 4	51.2	24 ± 3	20.7	25 ± 3	10.2	25 ± 3	17.3	23 ± 3	30.4
H48	Tnf	31 ± 2	0.3	30 ± 2	0.4	30 ± 2	0.4	30 ± 1	0.6	29 ± 1	0.5
H49	Tnfsf10	28 ± 2	2.8	28 ± 2	1.5	27 ± 1	2.6	27 ± 1	3.3	26 ± 1	3.7
H50	Ulk2	30 ± 1	0.9	29 ± 1	1.0	29 ± 1	0.9	29 ± 1	1.1	28 ± 1	1.0
H51	Vegf	38 ± 7	0.0	31 ± 12	0.2	28 ± 14	1.7	33 ± 12	0.0	33 ± 12	0.0
H52	Zfand5	27 ± 2	5.3	27 ± 1	2.7	27 ± 1	2.5	27 ± 1	2.5	27 ± 1	3.5
H53	Zfp36/Ttp	29 ± 2	1.9	29 ± 2	0.7	29 ± 2	0.6	29 ± 2	0.9	28 ± 2	1.2
H54	Zfp36l1	30 ± 3	1.2	30 ± 4	0.3	33 ± 7	0.1	31 ± 2	0.2	29 ± 5	0.8
H55	Zfp36l2	42 ± 5	0.0	35 ± 13	0.0	33 ± 11	0.0	33 ± 13	0.1	32 ± 12	0.1

The mean and standard deviation was calculated from 24 samples except DMSO (n = 12). The fold was calculated using the mean data and Bcl2 as the internal reference.

The mRNA level of a gene at least twofold or less than 50% of BCL2 mRNA could be interpreted as its expression more or less abundant than that of BCL2 mRNA, respectively. By this standard, 14 genes were expressed more abundantly than BCL2 gene (BCL2L1, BNIP3, CSNK2A1, CTSB, GAPDH, GLUT1, GLUT3, HIF1A, HMGR, IL6, MAP1LC3B, RPL32, TNFSF10, and ZFAND5) (Table 2). Similarly, 20 genes were expressed less abundantly than BCL2 gene (AHRR1, COX1, CXCL1, CYCLIND1, DGAT2A, DGAT2B, FAS, GLUT4, HUA, ICAM1, IL2, IL10, IL12, LEPTIN, NFKB, P53, RAB24, TNF, VEGF, and ZFP36L2) (Table 2). TaqMan qPCR assay showed similar trend of SYBR Green qPCR (data not shown). SYBR Green qPCR assay was chosen to conduct gene expression analysis in the following experiments.

### Selection of reference gene for qPCR assays in human colon cancer cells

Another important factor for comparing gene expression is to identify reference gene for qPCR analysis. Reference gene mRNA levels for qPCR assays should be minimally variable under experimental treatments. The C_T_ values with smaller standard deviations among the treatments indicate more stable gene expression. The qPCR data from 24 samples (triplicate each of the 8 concentrations: 0, 5, 10, 20, 30, 40, 50 and 100 µg/mL of ethanol extracts) were pooled and calculated for the mean ± standard deviation (Table 2). BCL2 C_T_ value was among the least varied with 29 ± 1, 29 ± 1, 29 ± 1, 28 ± 1 for glanded coat, glanded kernel, glandless coat, and glandless kernel extracts, respectively (mean ± standard deviation, n = 24) (Table 2). GAPDH and RPL32 are well-known reference genes for qPCR assays in mammalian cells. However, their C_T_ values had much larger standard deviations. GAPDH C_T_ value was among the largest variable in the cells with 25 ± 3, 25 ± 3, 25 ± 3, 23 ± 3 for glanded coat, glanded kernel, glandless coat, and glandless kernel extracts, respectively (mean ± standard deviation, n = 24) (Table 2). RPL32 C_T_ value was also among the largest variable in the cells with 24 ± 3, 25 ± 3, 25 ± 3, 23 ± 3 for glanded coat, glanded kernel, glandless coat, and glandless kernel extracts, respectively (mean ± standard deviation, n = 24) (Table 2). Furthermore, GAPDH and RPL32 mRNAs were the most abundant mRNAs among the 55 tested targets in the cells. The relative fold of GAPDH mRNA to BCL2 mRNA was 18, 10, 19, and 39 fold for glanded coat, glanded kernel, glandless coat, and glandless kernel extracts, respectively (n = 24) (Table 2). The relative fold of RPL32 mRNA to BCL2 mRNA was 21, 10, 17, and 30 fold for glanded coat, glanded kernel, glandless coat, and glandless kernel extracts, respectively (n = 24) (Table 2). They were much higher than the other mRNAs (Table 2). These data suggested that GAPDH and RPL32 mRNAs were not suitable internal references for qPCR assays in the human colon cancer cells due to large standard deviations and high expression levels. BCL2 mRNA was selected as the internal reference for our qPCR analyses since BCL2 was widely studied and least regulated gene in colon cancer cells.

There were 10 genes with mRNA levels at least twofold of BCL2 mRNA in the 24 pooled samples treated with glanded coat extract (BCL2L1, BNIP3, CSNK2A1, GAPDH, GLUT3, HIF1A, LEPTIN, MAP1LC3B, RPL32, and ZFAND5) (Table 2). There were 8 genes with mRNA levels at least twofold of BCL2 mRNA in the 24 pooled samples treated with glanded kernel extract (BNIP3, CSNK2A1, GAPDH, GLUT3, MAP1LC3B, RPL32, TNFSF10, and ZFAND5) (Table 2). There were 9 genes with mRNA levels at least twofold of BCL2 mRNA in the 24 pooled samples treated with glandless coat extract (BNIP3, CSNK2A1, GAPDH, GLUT1, GLUT3, MAP1LC3B, RPL32, TNFSF10, and ZFAND5) (Table 2). There were 13 genes with mRNA levels at least twofold of BCL2 mRNA in the 24 pooled samples treated with glandless kernel extract (BCL2L1, BNIP3, CSNK2A1, GAPDH, GLUT1, GLUT3, HIF1A, HMGR, LEPTIN, MAP1LC3B, RPL32, TNFSF10, and ZFAND5) (Table 2).

There were 23 genes with mRNA levels less than 50% of BCL2 mRNA in cells treated with glanded coat extract (AHRR1, CLAUDIN1, COX1, CXCL1, CYCLIND1, DGAT2A, DGAT2B, ELK1, FAS, GLUT4, HUA, ICAM1, INSR, IL2, IL10, IL12, NFKB, P53, RAB24, TNF, VEGF, ZFP36L1, and ZFP36L2) (Table 2). There were 24 genes with mRNA levels less than 50% of BCL2 mRNA in cells treated with glanded kernel extract (AHRR1, CLAUDIN1, COX1, CXCL1, CYCLIND1, DGAT1, DGAT2A, DGAT2B, ELK1, FAS, GLUT4, HMOX1, HUA, ICAM1, INSR, IL2, IL10, IL12, LEPTIN, NFKB, P53, RAB24, TNF, ZFP36L1, and ZFP36L2) (Table 2). There were 22 genes with mRNA levels less than 50% of BCL2 mRNA in cells treated with glandless coat extract (AHRR1, CLAUDIN1, COX1, CXCL1, CYCLIND1, DGAT2A, DGAT2B, ELK1, FAS, GLUT4, HUA, ICAM1, INSR, IL2, IL12, LEPTIN, NFKB, P53, RAB24, VEGF, ZFP36L1, and ZFP36L2) (Table 2). There were 21 genes with mRNA levels less than 50% of BCL2 mRNA in cells treated with glandless kernel extract (AHRR1, COX1, CXCL1, CYCLIND1, DGAT2A, DGAT2B, FAS, GLUT4, HMOX1, HUA, ICAM1, INSR, IL2, IL10, IL12, NFKB, P53, RAB24, TNF, VEGF, and ZFP36L2) (Table 2).

### Overall effect of cottonseed ethanol extracts on gene expression in human colon cancer cells

After we analyzed the basal levels of gene expression and identified the reference gene for qPCR analysis as described previously, we evaluated how these genes might be affected by ethanol extracts by using the pooled qPCR data from 24 samples using BCL2 mRNA as the internal reference and DMSO treatment as the sample control. As shown in Table 3, expression of a number of genes was affected by cottonseed ethanol extracts. There were 3 genes with mRNA levels at least twofold of the DMSO control in the cells treated with glanded coat extract (CYCLIND1, CYP19A1, and LEPTIN) (Table 3). There were 2 genes with mRNA levels at least twofold of the DMSO control in the cells treated with glanded kernel extract (CYCLIND1 and CYP19A1) (Table 3). There were 2 genes with mRNA levels at least twofold of the DMSO control in the cells treated with glandless coat extract (CYCLIND1 and CYP19A1) (Table 3). There were 4 genes with mRNA levels at least twofold of the DMSO control in the cells treated with glandless kernel extract (COX2, CYCLIND1, CYP19A1, and LEPTIN) (Table 3).

**Table 3:** Effect of cottonseed extracts on mRNA levels of 55 genes.

ID	mRNA	DMSO control	Glanded coat	Glanded kernel	Glandless coat	Glandless kernel
H1	Ahrr1	1	0.48	0.68	0.42	1.58
H2	Bcl2	1	1.00	1.00	1.00	1.00
H3	Bcl2l1	1	0.82	0.56	0.58	0.85
H4	Bnip3	1	0.75	0.66	0.81	0.82
H5	Cd36	1	0.53	0.70	0.65	0.85
H6	Claudin1	1	0.28	0.16	0.34	1.10
H8	Cox2	1	1.36	1.06	1.66	1.96
H9	Csnk2a1	1	0.42	0.34	0.40	0.60
H10	Ctsb	1	0.56	0.37	0.47	0.87
H11	Cxcl1	1	0.13	0.07	0.09	0.13
H12	Cyclind1	1	3.96	1.98	11.55	3.48
H13	Cyp19a1	1	14.99	13.37	15.32	12.74
H14	Dgat1	1	0.61	0.47	0.52	0.56
H15	Dgat2a	1	0.45	0.40	0.40	0.74
H16	Dgat2b	1	0.59	0.38	0.83	0.91
H17	E2f1	1	0.91	0.71	1.02	0.85
H18	Elk1	1	0.53	0.25	0.31	2.22
H19	Fas	1	0.83	0.43	1.77	1.62
H20	Gapdh	1	0.54	0.32	0.59	1.21
H21	Glut1	1	0.32	0.27	0.57	0.63
H22	Glut2	1	0.52	0.67	0.67	0.81
H23	Glut3	1	1.33	1.34	1.66	1.53
H25	Hif1a	1	0.87	0.71	0.67	1.39
H26	Hmgr	1	0.42	0.48	0.53	0.87
H27	Hmox1	1	0.88	0.54	0.70	0.33
H28	Hua	1	0.96	0.57	0.84	0.46
H29	Icam1	1	0.09	0.46	0.18	0.36
H31	Insr	1	0.19	0.25	0.42	0.39
H32	Il2	1	0.71	0.83	0.80	0.65
H33	IL6	1	0.54	0.64	0.67	0.96
H34	IL8	1	0.96	0.85	0.98	0.96
H37	Il16	1	0.35	0.48	0.49	0.69
H38	Il17	1	0.52	0.54	0.57	0.63
H39	Leptin	1	4.82	0.24	0.45	3.18
H40	Map1lc3a	1	0.75	0.56	0.85	0.79
H41	Map1lc3b	1	0.38	0.40	0.49	0.50
H42	Nfkb	1	0.10	0.00	0.01	0.22
H43	P53	1	0.39	0.44	0.59	1.10
H44	Pim1	1	0.50	0.55	0.64	0.63
H45	Pparr	1	0.94	0.85	0.90	1.08
H47	Rpl32	1	0.70	0.34	0.58	1.03
H48	Tnf	1	1.13	1.21	1.60	1.34
H49	Tnfsf10	1	0.65	1.11	1.40	1.58
H50	Ulk2	1	0.76	0.75	0.83	0.80
H52	Zfand5	1	0.63	0.60	0.59	0.83
H53	Zfp36/Ttp	1	0.51	0.44	0.65	0.87
H54	Zfp36l1	1	0.44	0.08	0.26	1.03

The fold was calculated using the mean C_T_ data, Bcl2 as the internal reference and DMSO as the sample control. Inos was undetectable and Cox1, Glut4, Il10, Il12, Rab24, Vegf and Zfp36l2 mRNAs were not analyzed due to their too low C_T_ values.

There were 13 genes with mRNA levels less than 50% of the DMSO control in the cells treated with glanded coat extract (AHRR1, CLAUDIN1, CSNK2A1, CXCL1, DGAT2A, GLUT1, HMGR, ICAM1, INSR, IL16, NFKB, P53, and ZFP36L1) (Table 3). There were 22 genes with mRNA levels less than 50% of the DMSO control in the cells treated with glanded kernel extract (CLAUDIN1, CSNK2A1, CTSB, CXCL1, DGAT1, DGAT2A, DGAT2B, ELK1, FAS, GAPDH, GLUT1, HMGR, ICAM1, INSR, IL16, LEPTIN, MAP1LC3B, NFKB, P53, RPL32, ZFP36, and ZFP36L1) (Table 3). There were 13 genes with mRNA levels less than 50% of the DMSO control in the cells treated with glandless coat extract (AHRR1, CLAUDIN1, CSNK2A1, CTSB, CXCL1, DGAT2A, ELK1, ICAM1, INSR, IL16, LEPTIN, MAP1LC3B, and NFKB) (Table 3). There were 6 genes with mRNA levels less than 50% of the DMSO control in the cells treated with glandless kernel extract (CXCL1, HMOX1, HUA, ICAM1, INSR, and NFKB) (Table 3).

The above results suggest that cottonseed ethanol extracts affected the expression of many genes in the human colon cancer cells. Therefore, we analyzed the mRNA levels of 55 genes in the human colon cancer cells treated with various concentrations of the four cottonseed extracts as described below.

### Effect of glanded coat extract on gene expression

**Figure 3:**
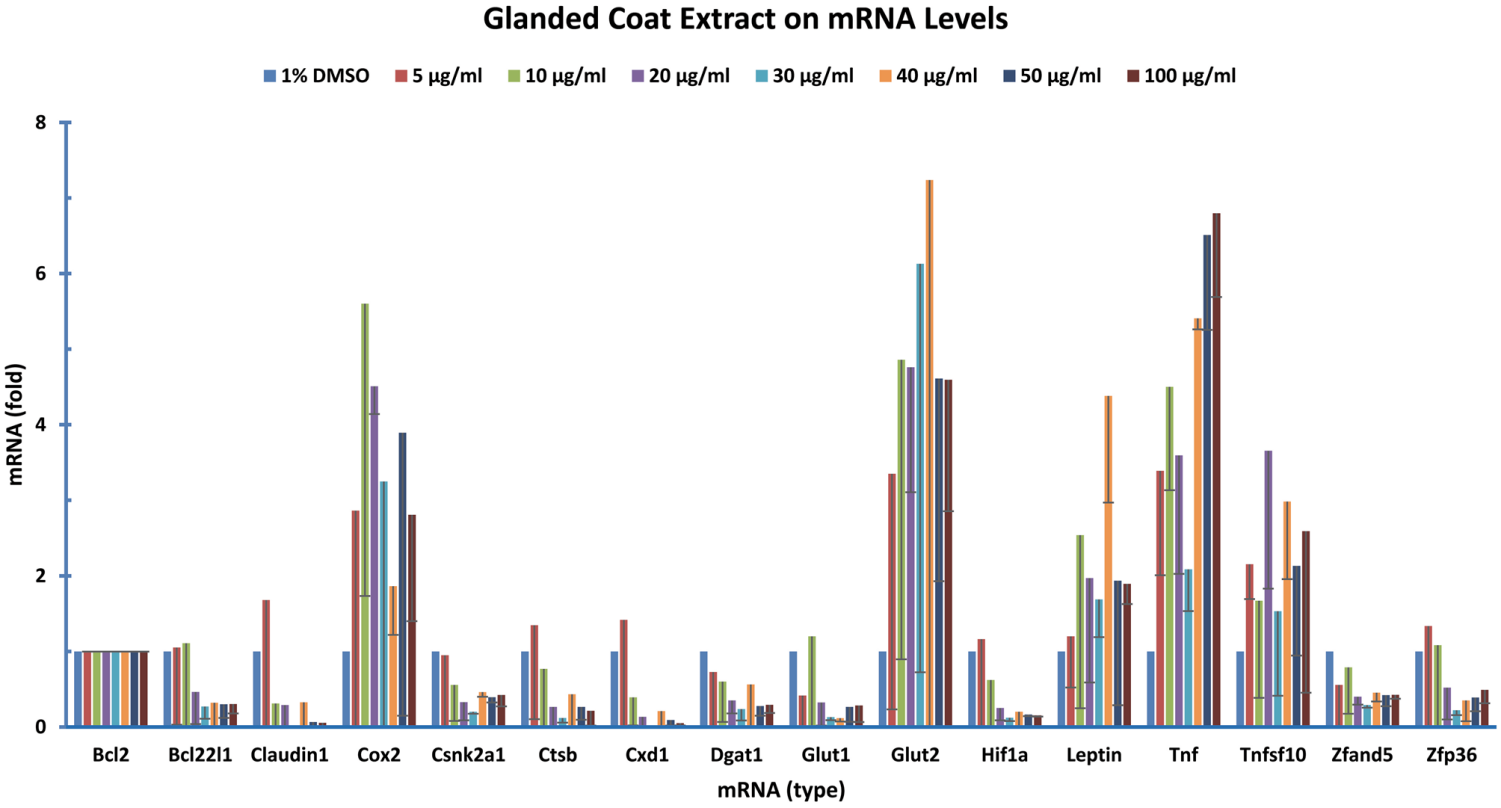
Glandless coat extract regulated the expression of genes. Human colon cancer cells (COLO 225) were treated with gossypol for 8 h. The data represent the mean and standard deviation of three independent samples.

Firstly, we analyzed the effect of glanded coat extract on gene expression. Human colon cancer cells were treated with glanded cottonseed coat extract (0, 5, 10, 20, 30, 40, 50 and 100 µg/ml). SYBR Green qPCR analyzed the expression of all 55 genes with BCL2 mRNA as the internal reference and 1% DMSO treatment as the sample control. The expression of some genes was significantly affected by glanded coat extract (Fig. 3). It appeared that the expression of COX2, GLUT1, LEPTIN, TNF, and TNFSF10 was increased by the glanded coat extract (Fig. 3). Other gene expression was reduced by the coat extract, including BCL22L2, CLUDIN1, CSNK2A1, CTSB, CXC1, DGAT1, GLUT1, HIF1, ZFAND5 and ZFP36 (Fig. 3). The expression of the rest of the 55 genes not mentioned above at mRNA levels was not affected by various concentrations of the glanded kernel extract (data not shown).

### Effect of glanded kernel extract on gene expression

**Figure 4:**
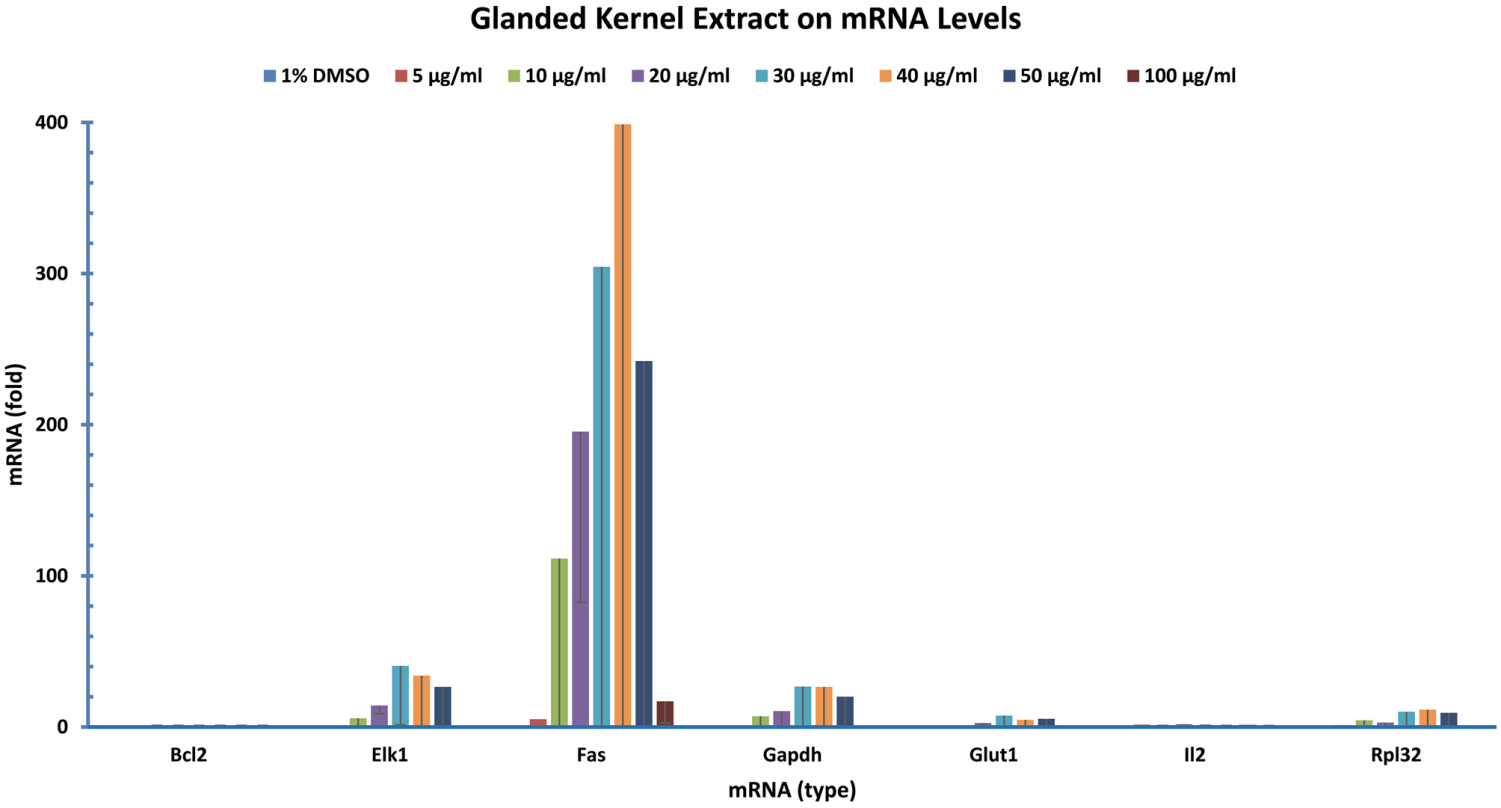
Glandless coat extract regulated the expression of genes. Human colon cancer cells (COLO 225) were treated with gossypol for 8 h. The data represent the mean and standard deviation of three independent samples.

Secondly, we analyzed the effect of glanded kernel extract on gene expression. Similarly, human colon cancer cells were treated with glanded cottonseed kernel extract. Gene expression was analyzed by qPCR with BCL2 mRNA as the internal reference and 1% DMSO treatment as the sample control. The expression of ELK1, FAS, and GAPDH genes was increased by the glanded kernel extract (Fig. 4). The expression of other genes at mRNA levels was not affected by various concentrations of the ethanol extract.

### Effect of glandless coat extract on gene expression

**Figure 5:**
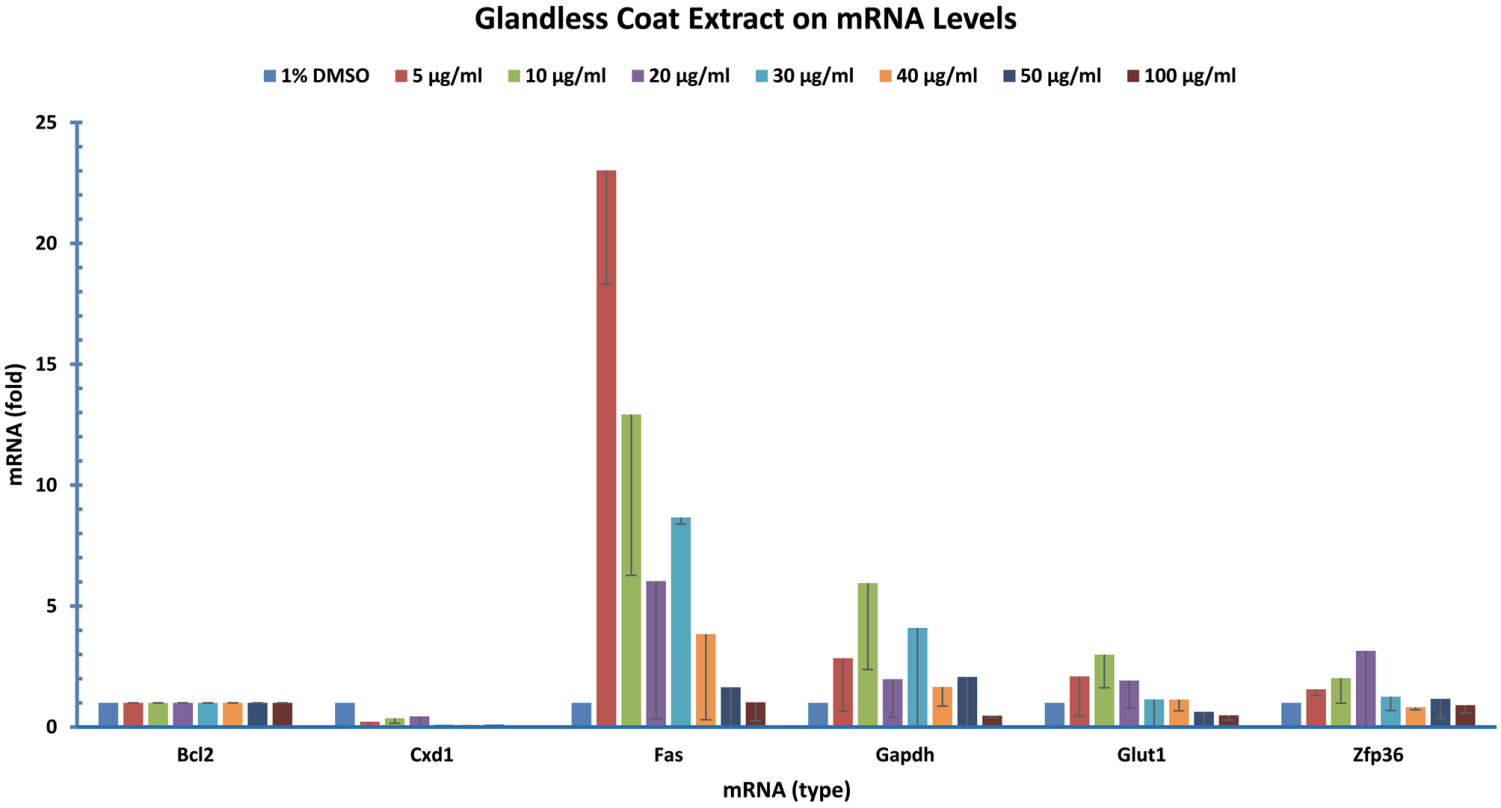
Glandless coat extract regulated the expression of genes in human colon cancer cells. Human colon cancer cells (COLO 225) were treated with gossypol for 8 h. The data represent the mean and standard deviation of three independent samples.

Thirdly, we analyzed the effect of glandless coat extract on gene expression. Human colon cancer cells were also treated with various concentrations of glandless cottonseed coat extract and analyzed gene expression at the mRNA levels by qPCR using BCL2 mRNA as the internal reference and 1% DMSO treatment as the sample control. The expression of FAS, GAPDH, GLUT1, and ZFP36 was increased by the glandless coat extract (Fig. 5), but only CXC1 expression was reduced by the coat extract (Fig. 5). The expression of the rest of the 55 genes not mentioned above at mRNA levels was not affected by various concentrations of the ethanol extract (data not shown).

### Effect of glandless kernel extract on gene expression

**Figure 6:**
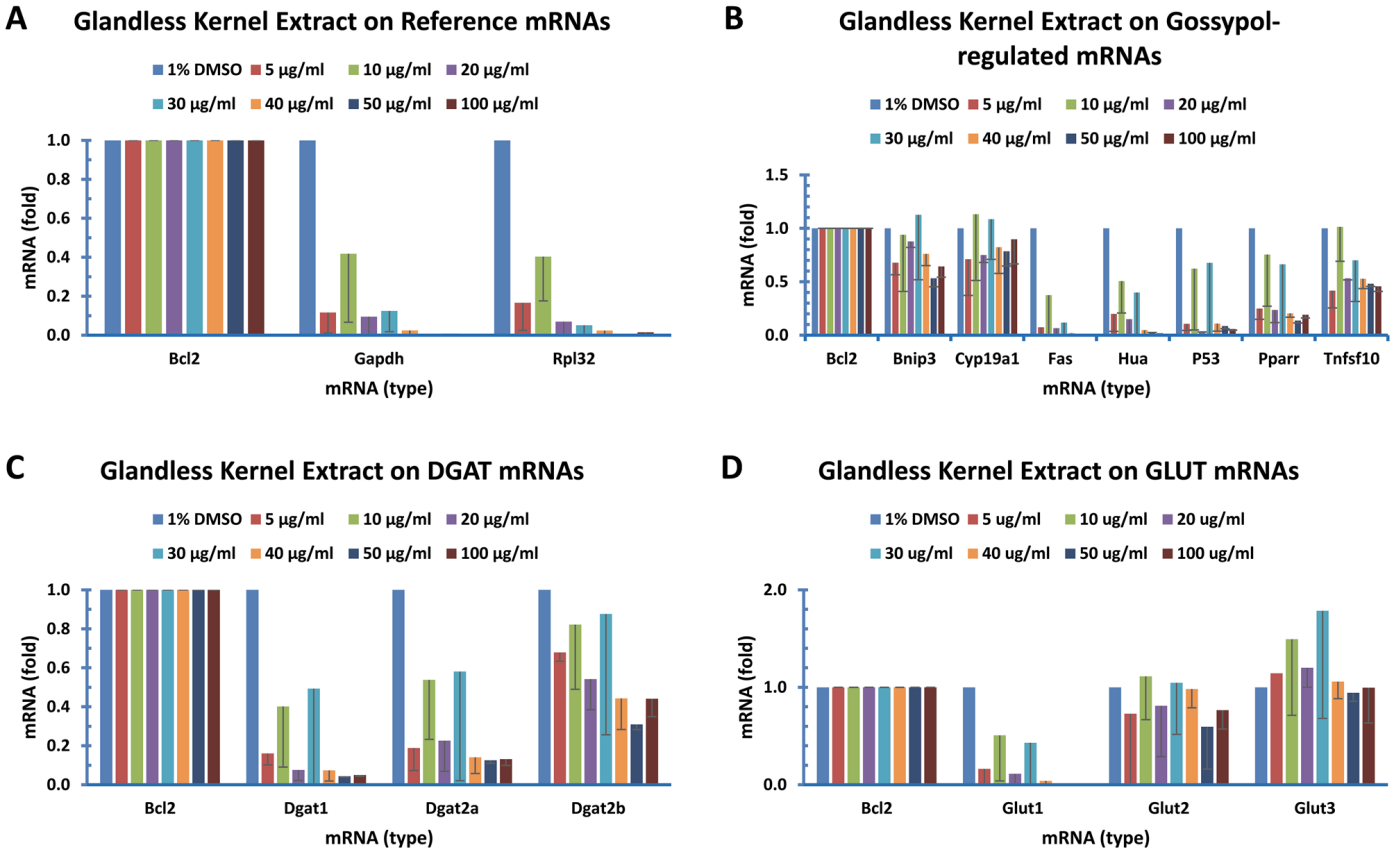
Glandless kernel extract regulated the expression of genes coded for qPCR reference mRNAs, genes reported to be regulated by gossypol, and genes coded for DGAT and GLUT mRNAs in human colon cancer cells. Human colon cancer cells (COLO 225) were treated with glandless kernel extract for 8 h. The data represent the mean and standard deviation of three independent samples. (**A**) Genes coded for qPCR reference mRNAs, (**B**) Genes reported to be regulated by gossypol, (**C**) Genes coded for DGAT mRNAs, (**D**) Genes coded for GLUT mRNAs.

**Figure 7:**
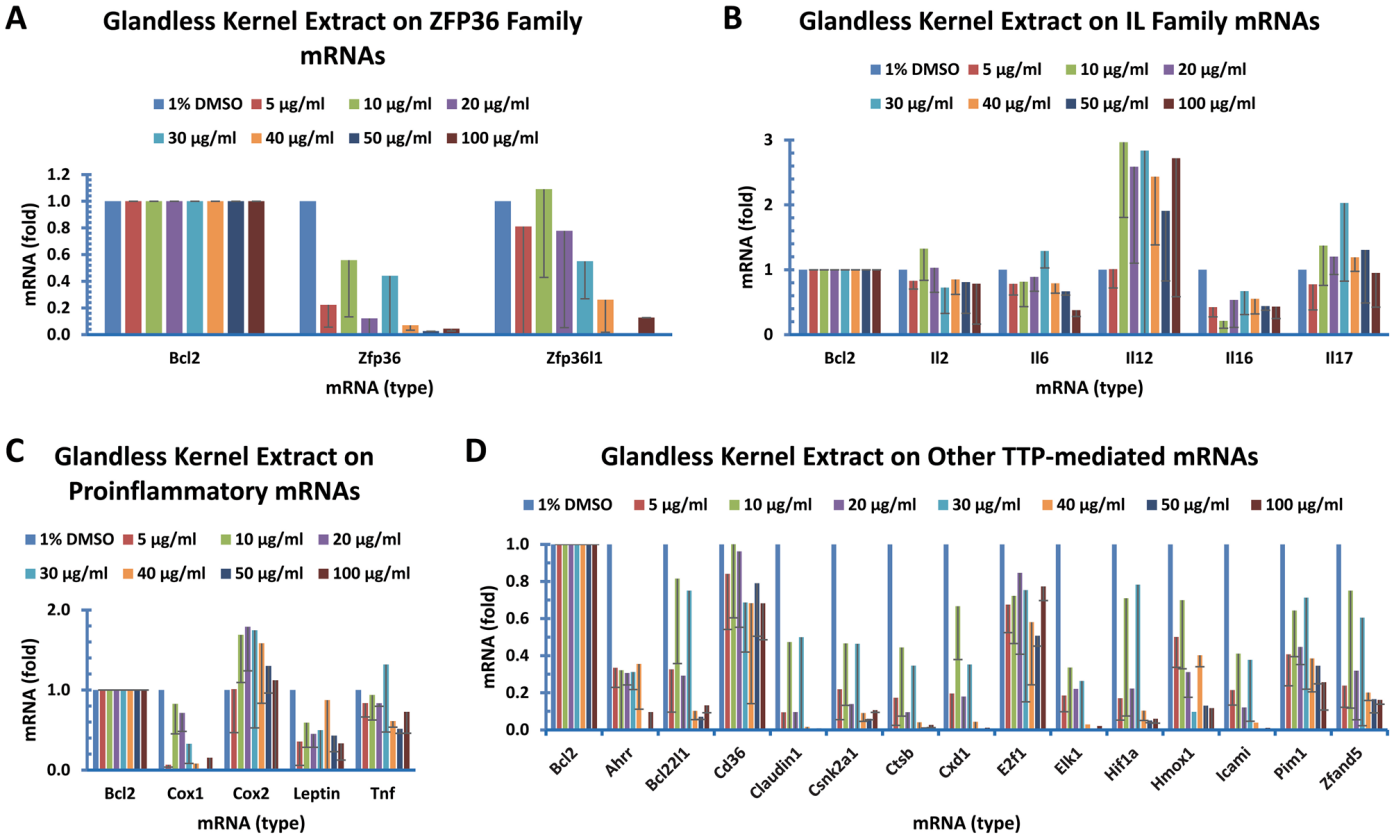
Glandless kernel extract regulated the expression of genes coded for TTP family, IL family, TTP-mediated proinflammatory cytokine and other mRNAs in human colon cancer cells. Human colon cancer cells (COLO 225) were treated with glandless kernel extract for 8 h. The data represent the mean and standard deviation of three independent samples. (**A**) Genes coded for TTP family mRNAs. (**B**) Genes coded for IL family mRNAs. (**C**) Genes coded for TTP-mediated proinflammatory cytokine mRNAs. (**D**) Genes coded for other TTP-mediated mRNAs.

**Figure 8:**
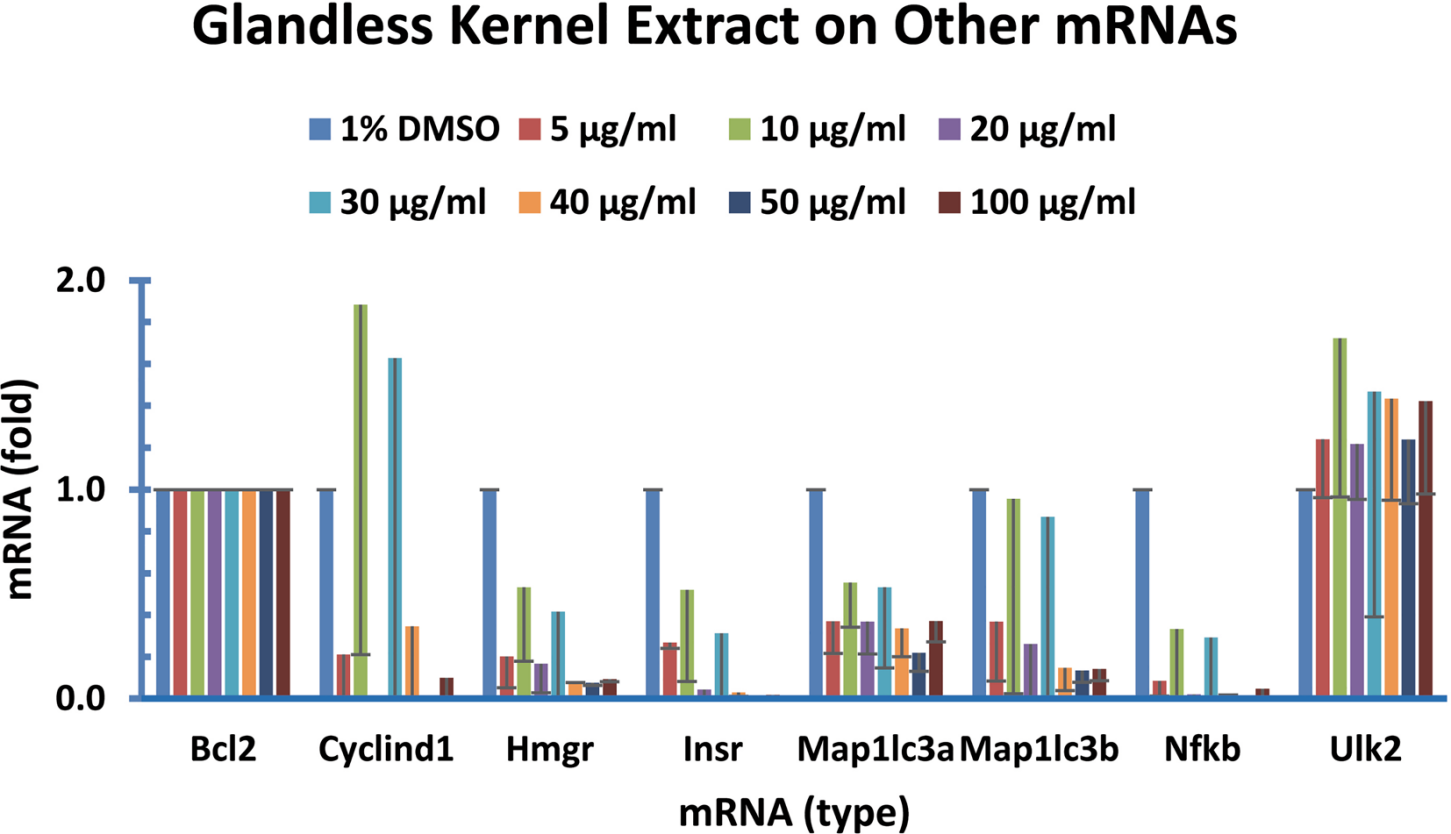
Glandless kernel extract regulated the expression of other genes in human colon cancer cells. Human colon cancer cells (COLO 225) were treated with glandless kernel extract for 8 h. The data represent the mean and standard deviation of three independent samples.

Finally, we analyzed the effect of glandless kernel extract on gene expression. Similarly, glandless cottonseed kernel extract treated human colon cancer cells and SYBR Green qPCR analyzed mRNA levels of 55 genes with BCL2 mRNA as the internal reference and 1% DMSO treatment as the sample control. qPCR data indicated that expression of much more genes was affected by the glandless kernel extract. The effect of the glandless kernel extract on gene expression was analyzed in detail according to gene families as described below (Figs. 6, 7, 8).

#### Glandless kernel extract on reference gene expression

The expression of GAPDH and RPL32 genes, the two well-known reference genes in the literature, was analyzed in the colon cancer cells after treatment with various concentration of glandless kernel extract. The qPCR data showed that glandless kernel extract treatment resulted in a large reduction of both GAPDH and RPL32 mRNA levels in the cells (Fig. 6A).

#### Glandless kernel extract on gossypol-related gene expression

Expression of several genes was regulated by gossypol in cancer cells^28–35^ and macrophages^26^. BNIP3, CYP19A1, FAS, HUA, P53, PPARR and TNFSF10 gene expression was analyzed in the colon cancer cells after being treated with glandless kernel extract with various concentrations. The expression of FAS, HUA, P53 and PPARR genes was inhibited to a large extent by the glandless kernel extract (Fig. 6B).

#### Glandless kernel extract on DGAT gene expression

Diacylglycerol acyltransferases (DGATs) catalyze the rate-limiting step of triacylglycerol biosynthesis in eukaryotes by esterifying*sn*-1,2-diacylglycerol with a long-chain fatty acyl-CoA^54,55^. DGATs are classified with DGAT1 and DGAT2 subfamilies in animals and additional DGAT3 subfamily in plants^54–57^ with DGAT2 mRNA being the major form of DGAT mRNAs in mouse adipocytes and macrophages^25,58^ but DGAT1 as the major one in the colon cancer cells^53^. The qPCR data showed that glandless kernel extract inhibited DGAT1, 2a and 2b expression in the human colon cancer cells (Fig. 6C).

#### Glandless kernel extract on GLUT gene expression

Glucose transporter (GLUT) family proteins are responsible for glucose uptake in mammalian cells. Four forms of GLUTs are present in mammalian cells^23^. The glandless kernel extract treatment only decreased GLUT1 mRNA level without much effect on the other GLUT isoforms (Fig. 6D). GLUT4 mRNA level was very low so that it was difficult to be measured with sufficient confidence (Table 2).

#### Glandless kernel extract on TTP gene expression

Tristetraprolin (TTP/ZFP36) family proteins regulate mRNA stability^59^. TTP family genes have anti-inflammatory properties with therapeutic potential for inflammation-related diseases^60,61^. TTP family proteins consist of three members in mammals (ZFP36 or TTP, ZFP36L1 and ZFP36L2) and the fourth member in mouse and rat but not in humans (ZFP36L3)^59,62^. SYBR Green qPCR showed that ZFP36 and ZFP36L1 mRNAs were reduced by the glandless kernel extract (Fig. 7A). ZFP36L2 mRNA levels were too low to be assessed reliably (Table 2).

#### Glandless kernel extract on IL gene expression

Several interleukins (ILs) are regulated by TTP family proteins which bind to AU-rich elements (ARE) of IL mRNAs and destabilizes the transcripts. TTP-regulated ILs include IL2^63^, IL6^64^, IL8^65^, IL10^66^, IL12^67^, IL16^42^ and IL17^68^. SYBR Green qPCR showed that glandless kernel extract increased IL12 mRNA level but decreased IL16 mRNA level (Fig. 7B). IL8 and IL10 mRNA levels were difficult to compare due to their low levels in the colon cancer cells (Table 2).

#### Glandless kernel extract on proinflammatory gene expression

Several proinflammatory cytokine mRNAs are destabilized by TTP family proteins, including tumor necrosis factor-alpha (TNFα)^60,69–71^, granulocyte–macrophage colony-stimulating factor/colony-stimulating factor 2 (GM-CSF/CSF2)^72,73^ and cyclooxygenase 2/prostaglandin-endoperoxide synthase 2 (COX2/PTGS2)^43^. TNFα and GM-CSF mRNAs are stabilized in TTP knockout mice and in cells derived from them^60,73^, resulting in excessive levels of these cytokines causing a severe systemic inflammatory syndrome including arthritis, autoimmunity, and myeloid hyperplasia^74,75^. Elevated levels of TTP reduce inflammatory responses in macrophages^76^. These previous studies suggest that TTP is an anti-inflammatory protein. Our results showed that glandless kernel extract decreased COX1, LEPTIN and TNF mRNA levels in the colon cancer cells (Fig. 7C).

#### Glandless kernel extract on TTP-targeted other gene expression

Other TTP-regulated mRNAs have been reported in the literature (Table 1). SYBR Green qPCR analyzed the mRNA levels of AHRR, BCL22L1, CD36, CLAUDIN1, CSNK2A1, CTSB, CXD1, E2F1, ELK1, HIF1A, HOMX1, ICAMI, PIM1, and ZFAND5 genes. Glandless kernel extract decreased all of these TTP-targeted mRNA levels except CD36 and E2F1 mRNA levels (Fig. 7D).

#### Glandless kernel extract on other gene expression

A few other gene targets were selected for the analysis of gene expression. The qPCR assays showed that glandless kernel extract decreased the expression of HMGR, INSR, MAPL1C3A, MAPL1C3B, and NFKB mRNA levels (Fig. 8). The effect of glandless kernel extract on ULK2 mRNA level was not much and the effect on CYCLIND1 mRNA level was difficult to assess due to large variation of the results (Fig. 8).

## Discussion

Cottonseed accounts for approximately 20% of the crop value. One way to increase cottonseed value is to isolate bioactive materials aimed at improving nutrition and preventing diseases. In this study, we observed that the expression of the majority of genes was significantly reduced by glandless cottonseed kernel extract, although their expression was less affected by three other cottonseed ethanol extracts (glanded cottonseed coat and kernel as well as glandless cottonseed coat extracts).

Cottonseed extracts exhibited only minor effect on the viability of human colon cancer cells under the experimental conditions. Our previous study showed that gossypol strongly inhibited human cancer cell viability^24^. The current data confirm our HPLC–MS analyses that the cottonseed extracts are essentially free of the toxic compound gossypol^24^.

Before we examined the effect of cottonseed extracts on gene expression in human colon cancer cells, we evaluated the relative expression levels of 55 genes and selected the internal reference for qPCR analysis since it is important for normalization of gene expression levels^77–80^. Our study confirmed that BCL2 mRNA was the most stable among the 55 mRNAs analyzed in human colon cancer cells treated with DMSO vehicle or various concentrations of ethanol extracts (Table 2)^53^. We also confirmed that GAPDH and RPL32 mRNAs were not good qPCR assay references for the colon cancer cells since they were most abundant mRNAs with large variations under the cell culture conditions^53^.

Our study showed that expression of many genes in human colon cancer cells was somewhat affected by cottonseed ethanol extracts. Although extracts isolated from glanded seed coat and kernel as well as glandless seed coat showed less effects on gene regulation, the expression of the majority of genes was significantly reduced by glandless seed kernel extract (Figs. 4, 5, 6, 7, 8). qPCR analyses showed that glanded coat extract increased COX2, GLUT2, LEPTIN, TNF, and TNFSF10 but decreased BCL22L2, CLUDIN1, CSNK2A1, CTSB, CXC1, DGAT1, GLUT1, HIF1, ZFAND5 and ZFP36 mRNA levels (Fig. 3). Glanded kernel extract increased ELK1, FAS, and GAPDH mRNA levels (Fig. 4). Glandless coat extract increased FAS, GAPDH, GLUT1, and ZFP36 but decreased CXC1 mRNA levels (Fig. 5).

The most important observation of this study was that glandless kernel extract decreased the mRNA levels of the great majority of the 55 genes tested, including GAPDH involved in the sixth step of breakdown of glucose in glycolysis^80^ and RPL32, a component of the large 60S subunit of ribosomes involved in protein synthesis^77^ (Fig. 6A), the genes known to be involved in cancer development, such as BNIP3 involved in the permeability of outer mitochondrial membrane^35^, CYP19A1 localized to the endoplasmic reticulum and catalyzed the last steps of estrogen biosynthesis^30^, FAS, a member of TNF-receptor superfamily playing a key role in programmed cell death^29^, P53 involved in preventing genome mutation^28^, PPARR, a nuclear receptor involved in gene expression regulation^31^ and TNFSF10, a TNF super family member functioning as a ligand that induces apoptosis^34^ (Fig. 6B), the DGAT family members DGAT1, DGAT2a and 2b responsible for the last and rate-limiting step of triacylglycerol biosynthesis^54,58^ (Fig. 6C), and GLUT1 responsible for glucose transport across the plasma membranes^23^ (Fig. 6D). In addition, glandless kernel extract reduced ZFP36 mRNA levels in the TTP family which bind to the AU-rich elements of some mRNAs and cause destabilization^60,69^ (Fig. 7A). It increased IL12, a T-cell stimulating fsctor^67^ but decreased IL16 functions as a chemoattractant, a modulator of T cell activation, and an inhibitor of HIV replication^42^ mRNAs levels in the IL family members (Fig. 7B), decreased LEPTIN involved in energy balance^81^ and TNF, a cytokine promoting inflammation^64^ mRNA levels (Fig. 7C), and appeared to decrease all of the TTP-targeted mRNAs including AHRR1^39^, BCL2L1^82^, CSNK2A1^41^, CXCL1^83^, HIF1a^84^, E2F1^40^, ELK1^37^, HMOX1^85^, ICAM1^86^ and ZFAND5^87^ (Fig. 7D). Finally, glandless kernel extract appeared to decrease the expression of HMGR^88^, INSR^21^, MAPL1C3A^89^, MAPL1C3B^89^, and NFKB^90^ mRNA levels (Fig. 8).

This study provides valuable information about the effects of cottonseed ethanol extracts on gene expression at the mRNA levels in the human colon cancer cells. Much more investigations need to be conducted in the future. First, it could be a greater addition by confirming the mRNA results with results at the protein levels. Second, the consequence of gene regulation on cellular metabolic levels could be valuable for understanding the molecular mechanism. Finally, additional studies with other cell lines and animals could be required for the potential utilization of cottonseed extracts as viable sources for improving nutrition and preventing diseases.

## Conclusions

This study showed that most of the gene expression in human colon cancer cells was not affected by ethanol extracts isolated from glanded cottonseed coat and kernel as well as glandless cottonseed coat, but the expression of the majority of genes was significantly reduced by glandless cottonseed kernel extracts. The inhibitory effects of glandless kernel extract on gene expression in the colon cancer cells may provide a useful opportunity for improving the healthcare associated with colon cancer since it is safe without toxic gossypol contamination and effective in decreasing the expression of so many genes related to cancer development. This in turn may provide the potential of increasing the value of cottonseed by using cottonseed-derived ethanol extracts as a health intervention agent.

## Materials and methods

### Cottonseed

The cottonseeds used in the study were provided by Drs. Michael Dowd and Rick Byler (USDA-ARS) and Tom Wedegaertner (Cotton, Inc.). The experiments were performed in accordance with national/institutional guidelines and regulations.

### Cancer cell line

Human colon cancer cells (COLO 205) and A549 lung cancer cells (CCL185) (ATCC, Manassas, VA) were kept under liquid nitrogen vapor. The cells were maintained in a humidified incubator at 37 °C with 5% CO_2_ in RPMI-1640 (COLO 205) and F-12K (CCL185) medium, respectively, supplemented with 10% (v:v) fetal bovine serum, 0.1 million units/L penicillin, 100 mg/L streptomycin, and 2 mmol/L L-glutamine (Gibco, Life Technologies).

### Chemicals, reagents and equipment

Cell cytotoxicity reagent (MTT based-In Vitro Toxicology Assay Kit) and DMSO were from Sigma. Tissue culture reagents were from Gibco BRL (Thermo Fisher). Tissue culture incubator was water jacket CO_2_ incubator (Thermo Fisher). Tissue culture workstation was Logic + A2 hood (Labconco, Kansas City, MO). Tissue culture plastic ware was from CytoOne (USA Scientific, Ocala, FL). Cell counting reagent (trypsin blue dye), slides (dual chamber), counter (TC20 Automatic Cell Counter) and microscope (Zoe Florescent Cell Imager) were from Bio-Rad (Hercules, CA). Microplate spectrophotometer (Epoch) was from BioTek Instruments (Winooski, VT).

### Cottonseed extracts

Seed kernel extracts were isolated by fractionation, defatting, and ethanol extraction, and seed coat extracts were isolated by fractionation, defatting, acetic acid extraction, and ethanol extraction^24^ (Fig. 1). Briefly, cottonseed coat or kernel was ground into fine powder and homogenized. The kernel fraction was defatted with chloroform and hexane. The coat fraction was treated with acetic acid followed by autoclave and centrifugation. The defatted materials were extracted with ethanol followed by evaporation to remove acetic acid and ethanol. Ethanol extracts were reconstitution in 100% DMSO (100 mg/mL) and analyzed by HPLC–MS. The ethanol extracts contained trace amount of gossypol (0.82 ng gossypol/mg extract in glanded seed coat, 0.03 ng gossypol/mg extract in glanded seed kernel, 0.37 ng gossypol/mg extract in glandless seed coat and 0 ng gossypol/mg extract in glandless seed kernel)^24^.

### Cell culture and chemical treatment

Cell culture was according to previous procedures^19,23,69^. Cancer cells were dissociated from flasks with 0.25% (w/v) trypsin-0.53 mM EDTA solution, stained with 0.2% trypsin blue dye and counted the number of live cells with a TC20 Automatic Cell Counter. Cells were subcultured at ~ 1 × 10^5^ cells/mL density in 24-well plates (0.5 mL). The cancer cells were routinely observed under a Zoe Florescent Cell Imager. Cancer cells were treated with 0, 5, 10, 20, 30, 40, 50 and 100 µg/mL of ethanol extracts for 2, 4, 8 and 24 h (“0” treatment as the vehicle control corresponding to 1% DMSO present in all of the culture medium).

### Cell viability assay

MTT based-In Vitro Toxicology Assay Kit was used to determine cell cytotoxicity^24^. Cancer cells in 96-well plates (12 wells/treatment) were treated with ethanol extracts and incubated at 37 °C, 5% CO_2_ for 2 and 24 h. The cell media were added with 50 µL of MTT assay reagent (thiazolyl blue tetrazolium bromide) and incubated at 37 °C, 5% CO_2_ for 2 h before adding 500 µL MTT solubilization solution to each well, shaken at room temperature overnight. The color density in the wells was recorded by Epoch microplate spectrophotometer at A570.

### Real-time qPCR primers and probes

Fifty-five genes were selected for qPCR analysis of their expression in the colon cancer cells as described previously^53^. These genes were shown to be regulated by cottonseed-derived gossypol in cancer cells and macrophages or regulated by ZFP36/TTP in tumor cells and macrophages (Table 2). RNA sequences were obtained from NCBI’s non-redundant protein sequence databases (http://blast.ncbi.nlm.nih.gov/Blast.cgi). The qPCR primers were designed with Applied Biosystems’ Primer Express software (Foster City, CA) and synthesized by Biosearch Technologies, Inc. (Navato, CA).

### RNA isolation and cDNA synthesis

RNA isolation and cDNA synthesis were essentially as described^25^. Human colon cancer cells were treated with various concentrations of cottonseed ethanol extracts for 8 h (triplicate). The cells were lysed directly in the washed dishes with 1 mL of TRI_ZOL_ reagent. RNA was isolated according to the manufacturer’s instructions without DNase treatment. RNA concentrations were quantified with an Implen NanoPhotometer (Munchen, Germany). The cDNAs were synthesized from total RNA using SuperScript II reverse transcriptase. The cDNA synthesis mixture contained 5 μg total RNA, 2.4 μg oligo(dT)^12–18^ primer, 0.1 μg random primers, 500 μM dNTPs, 10 mM DTT, 40 u RNaseOUT and 200 u SuperScript II reverse transcriptase in 1X first-strand synthesis buffer (20 μL). The cDNA synthesis reaction was performed at 42 °C for 50 min. The cDNA was stored in − 80 °C freezer and diluted with water to 1 ng/µL before qPCR analyses.

### Quantitative real-time PCR analysis

The qPCR assays were described^56,78,79,91^ and performed according to the MIQE guidelines: minimum information for publication of quantitative real-time PCR experiments^92^. The qPCR assay mixture contained 5 ng of RNA-derived cDNA, 200 nM of forward and reverse primers, and 1 × iQ SYBR Green Supermix. Thermal cycle conditions were 3 min at 95 °C, 40 cycles at 95 °C for 10 s, 65 °C for 30 s and 72 °C for 30 s. BCL2 mRNA was used as the internal reference because it had the minimal variation of gene expression among the 55 genes tested (see “Results” for details). Ribosome protein 32 (RPL32) and glyceraldehyde-3-phosphate dehydrogenase (GAPDH) mRNAs were not suitable for qPCR analysis for this cell type due to variations (see “Results” for details) although they were widely used as the reference mRNAs in qPCR analyses^19^. TaqMan qPCR assay confirmed some of the SBYR Green qPCR assays using the same conditions as described^78^.

### Data analysis and statistics

The relative expression in fold was determined with 2^−Δ*CT*^ or 2^−ΔΔ*CT*^ equations^93^. The first step was to normalize the threshold cycle (*C*_*T*_) values of the target mRNAs to the *C*_*T*_ values of the internal control BCL2 mRNA (Δ*C*_*T*_ = *C*_*T*Target_ − *C*_*T*Bcl2_). The second step was to normalize treatment Δ*C*_*T*_ values with DMSO control Δ*C*_*T*_ values (ΔΔ*C*_*T*_ = Δ*C*_*T*Cottonseed_ − Δ*C*_*T*DMSO_). Finally, the fold change in expression was calculated. The data in the figures and tables represent the mean and standard deviation of three and 24 independent samples, respectively. These data were subjected to statistical analysis using ANOVA with SigmaStat 3.1 software (Systat Software). Student–Newman–Keuls method and Tukey test were used to perform multiple comparisons among the treatments with different concentrations of cottonseed extracts^19^.
